# Endothelial and Macrophage-Specific Deficiency of P38α MAPK Does Not Affect the Pathogenesis of Atherosclerosis in ApoE^−/−^ Mice

**DOI:** 10.1371/journal.pone.0021055

**Published:** 2011-06-09

**Authors:** Rozina Kardakaris, Ralph Gareus, Sofia Xanthoulea, Manolis Pasparakis

**Affiliations:** Centre for Molecular Medicine, Institute for Genetics, Cologne Excellence Cluster on Cellular Stress Responses in Aging-Associated Diseases, University of Cologne, Cologne, Germany; Istituto Dermopatico dell'Immacolata, Italy

## Abstract

**Background:**

The p38α Mitogen-Activated Protein Kinase (MAPK) regulates stress- and inflammation-induced cellular responses. Factors implicated in the development of atherosclerosis including modified low-density lipoprotein (LDL), cytokines and even shear stress induce p38 activation in endothelial cells and macrophages, which may be important for plaque formation. This study investigates the effects of endothelial- and macrophage-specific deficiency of p38α in atherosclerosis development, in Apolipoprotein E deficient (*ApoE^−/−^*) mice.

**Methodology/Principal Findings:**

*ApoE* ^−/−^ mice with macrophage or endothelial cell-specific p38α deficiency were fed a high cholesterol diet (HCD) for 10 weeks and atherosclerosis development was assessed by histological and molecular methods. Surprisingly, although p38α-deficiency strongly attenuated oxidized LDL-induced expression of molecules responsible for monocyte recruitment in endothelial cell cultures in vitro, endothelial-specific p38α ablation in vivo did not affect atherosclerosis development. Similarly, macrophage specific deletion of p38α did not affect atherosclerotic plaque development in *ApoE* ^−/−^ mice.

**Conclusions:**

Although previous studies implicated p38α signaling in atherosclerosis, our in vivo experiments suggest that p38α function in endothelial cells and macrophages does not play an important role in atherosclerotic plaque formation in ApoE deficient mice.

## Introduction

Atherosclerosis is the primary underlying cause of cardiovascular disease and the major cause of mortality in the western world today [1], [2]. Originally considered a primarily metabolic disorder, atherosclerosis is now recognised as a complex disease with a strong inflammatory component [3]. Upon exposure to oxidized lipids, vascular endothelial cells are activated to express chemotactic factors and adhesion molecules that are crucial for the recruitment of monocytes. The recruited monocytes migrate into the sub-endothelial space and differentiate into macrophages that phagocytose lipids to become ‘foam cells’. Accumulation of foam cells beneath the endothelium, accompanied by the infiltration of T cells, leads to the formation of the ‘fatty streak’, which can later on progress into advanced atherosclerotic plaques and their complications [4]. Foam cells eventually die by apoptosis within the lesions and their clearance by other phagocytes is important to prevent their secondary necrosis and the formation of a necrotic core [5]. The mechanisms that control the recruitment, retention and cell survival, as well as the clearance of apoptotic foam cells within the lesions, are crucial for the pathogenesis of atherosclerosis. Pro-inflammatory signalling pathways that are activated downstream of innate immune and cytokine receptors, including the NF-κB and Mitogen-Activated Protein Kinase (MAPK) cascades, control immune, inflammatory and cell death responses and have been implicated in the pathogenesis of atherosclerosis [6], [7], [8].

The p38 MAPK pathway is crucial for a wide range of biological processes, including cell cycle, cell differentiation and apoptosis and the expression of inflammatory cytokines and chemokines [9]. p38α, the most physiologically relevant isoform of p38 involved in inflammatory responses [10], [11], has been implicated in the development and progression of atherosclerosis. Various roles have been attributed to this kinase with respect to atherogenesis, including the regulation of scavenger receptor expression and oxidized low-density lipoprotein (oxLDL) uptake by macrophages [12], control of the expression of the monocytic chemokine receptor CXCR2 that is induced in response to oxLDL [13] and the migration, proliferation [14], [15] permeability [16], apoptosis [17] and adhesion molecule expression (e.g. VCAM1) [18] of endothelial cells. Finally, p38α is critical for the production of multiple pro-inflammatory cytokines like tumor necrosis factor (TNF) and interleukins IL-1β, IL-6 and IL-8 in most of the cell types that participate in atherosclerosis development [18], [19], [20]. Thus, p38α has been suggested to have a crucial role in the pathogenesis of atherosclerosis by acting in cells that play a crucial role in atherogenesis, like endothelial cells, smooth muscle cells and macrophages.

Here, we used conditional gene targeting in mice to address *in vivo*, for the first time, the endothelial cell-specific function of p38α in atherosclerosis, in the apolipoprotein E deficient (*ApoE* ^−/−^) mouse model. Using the same model, we also studied the macrophage-specific role of p38α in this disease. We generated *ApoE* ^−/−^ mice lacking p38α in vascular endothelial cells or macrophages and analyzed the development of atherosclerotic plaques, after feeding a high cholesterol diet (HCD). Our results show that specific ablation of p38α in macrophages or endothelial cells did not affect the development of atherosclerotic plaques in *ApoE* ^−/−^ mice, suggesting that p38α activity in these cell types does not play a crucial role for the pathogenesis of atherosclerosis.

## Results

### P38α-deficiency inhibits oxLDL-induced adhesion molecule and chemokine expression in primary endothelial cells

**Figure 1:**
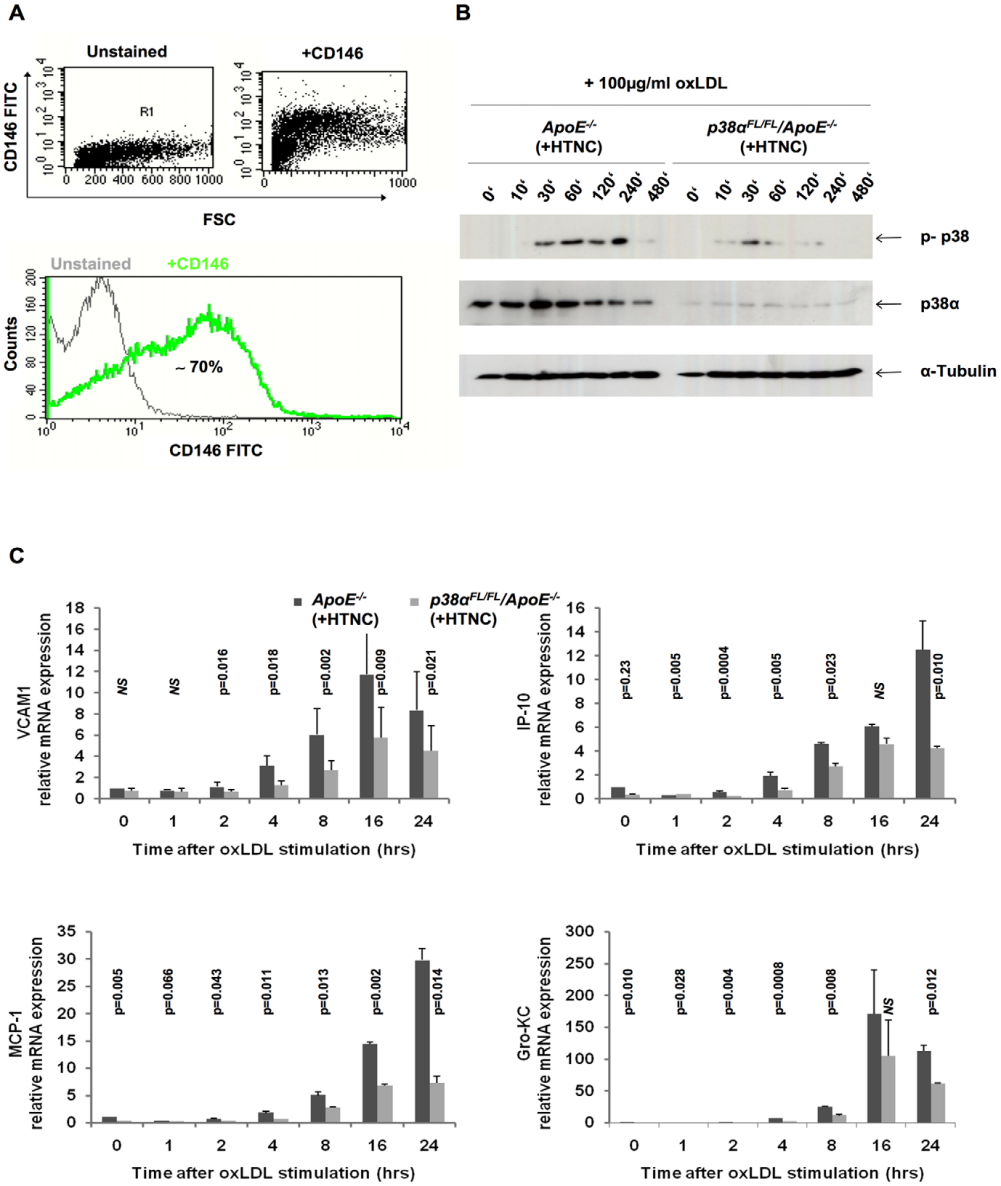
Attenuated monocyte recruitment molecule expression in p38α deficient endothelial cells, in vitro. (A) Flow cytometric analysis of primary lung endothelial cells to check for purity of population, after treatment with HTNC for 16 hrs. Cells were stained with CD146 FITC, specific for MLECs. Leukocytes isolated from blood were used as negative controls for CD146 staining (data not shown). (B) Immunoblotting for p38α and total p-p38 on MLEC protein extracts. (C) Relative mRNA expression levels of adhesion molecule VCAM1 and chemokines IP-10, MCP-1 and Gro-KC in MLECs. Cells were isolated from the lung of *p38α^FL/FL^/ApoE^−/−^* and *ApoE^−/−^* mice, passaged twice and treated with HTNC for 16 hrs to induce cre recombination. Data shown are representative of two separate experiments. Error bars represent SD.

Vascular endothelial cells have an important role in the pathogenesis of atherosclerosis by expressing adhesion molecules and chemokines that facilitate the recruitment of inflammatory cells into the developing plaque. To address the role of p38α in endothelial cells in atherosclerosis, we studied the contribution of p38α activation in the expression of adhesion molecules and chemokines by endothelial cells, upon stimulation with oxLDL. For these experiments, we used primary mouse lung endothelial cells (MLECs) isolated by Dynabead-mediated negative (LEAF) and positive (CD102) selection from dissociated lung tissue from *p38α^FL/FL^/ApoE^−/−^* or *ApoE^−/−^* control mice. To delete p38α in these cells, we used His-TAT-NLS-Cre (HTNC), a transducible Cre recombinase that can be used efficiently to mediate recombination of loxP flanked alleles in culture [21]. Purity of endothelial cell cultures after Cre recombination was assessed by flow cytometric analysis after staining with CD146 (Figure 1A). Immunoblot analysis with p38α specific antibodies revealed a strong reduction of p38α expression in HTNC-treated *p38α^FL/FL^/ApoE^−/−^* MLECs, indicating efficient ablation of p38α (Figure 1B, middle panel). Furthermore, immunoblot with antibodies recognizing phosphorylated p38 revealed that stimulation with 100 µg/ml oxLDL induced strong p38 activation in control (*ApoE^−/−^*) cells, while p38α-deficient MLECs showed weak p38 activation. As the phospho-p38 antibody used is not isoform-specific, the weak p38 phosphorylation detected most likely corresponds to phosphorylation of the residual p38α and also other p38 isoforms, such as p38β, which is the other major p38 isoform expressed in endothelial cells [22], (Figure 1B, top panel). We then assessed the oxLDL-induced expression of vascular adhesion molecule 1 (VCAM1) and the chemokines IP-10, MCP-1 and Gro-KC, known to be involved in the recruitment of monocytes into the arterial intima. Quantitative real-time PCR (qRT-PCR) analysis on RNA, isolated at different time points after oxLDL stimulation, revealed induction in expression of VCAM1, IP-10, MCP-1 and Gro-KC in control MLECs, which was strongly reduced in MLECs lacking p38α (Figure 1C).

### Endothelial cell p38α deficiency does not affect atherosclerosis development in ApoE^−/−^ mice

**Figure 2:**
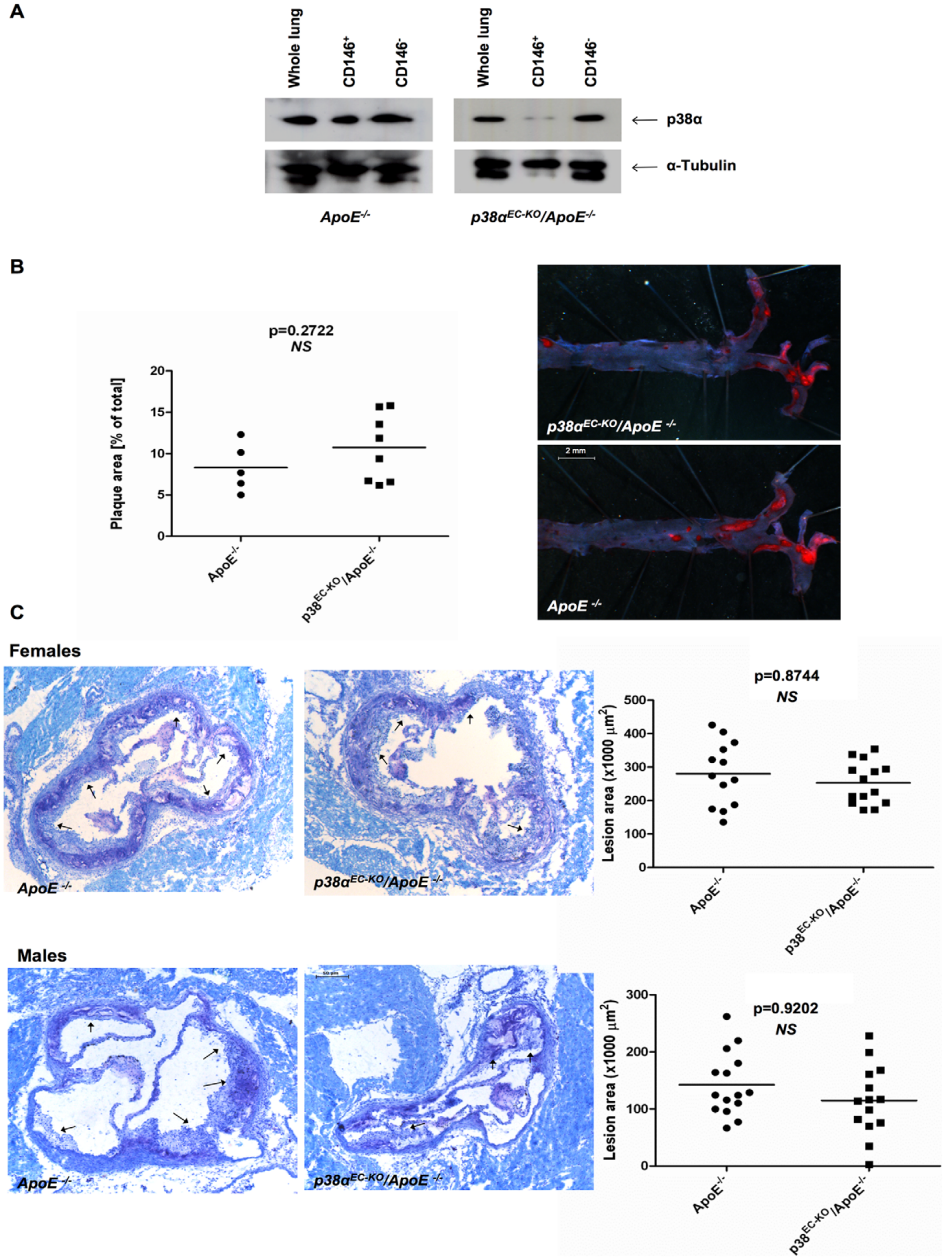
Comparable atherosclerotic plaque formation in *p38α^EC-KO^/ApoE^−/−^* and *ApoE^−/−^* mice after 10 weeks of HCD. (A) Analysis of deletion efficiency by immunoblotting in *p38α^EC-KO^/ApoE^−/−^* and their *ApoE* ^−/−^ littermates after 5 weeks of tamoxifen diet and 10 weeks of HCD, by MACS sorting of MLECs with CD146. CD146^+^ = MLECs, CD146^−^ = other cells. *P38^EC-KO^/Apo^−/−^* females, n = 9; *ApoE^−/−^* females, n = 6. (B) Quantification of lesion area on whole aorta from female *p38α^EC-KO^/ApoE^−/−^* and *ApoE^−/−^* mice. *En face* Sudan IV staining of plaques. *P38α^EC-KO^/ApoE^−/−^* females, n = 14; *ApoE* ^−/−^ females, n = 13. Scale bar, 2 mm. (C) Quantification of lesion area on atherosclerotic plaques at the aortic sinus of *p38α^EC-KO^/ApoE^−/−^* and *ApoE^−/−^* mice. Plaques are marked by arrows on aortal cross sections at the height of the aortic sinus. *P38α^EC-KO^/ApoE^−/−^* females, n = 14; *ApoE* ^−/−^ females, n = 13; *p38α^EC-KO^/ApoE^−/−^* males, n = 14; *ApoE* ^−/−^ males, n = 15. Scale bar, 0.2 mm.

The results of the above described experiments suggested that p38α activity in endothelial cells might play an important role for the development of atherosclerotic plaques and encouraged us to study *in vivo* the effect of endothelial cell specific p38α ablation, in the development of atherosclerosis. For this purpose we used Cre-loxP-mediated conditional targeting of p38α in ApoE-deficient mice, which spontaneously develop atherosclerotic plaques due to elevated blood cholesterol levels, a pathology that is further aggravated upon feeding with a HCD. Thus, we crossed mice carrying loxP-flanked p38α alleles with Tie2ER^T2^Cre transgenics, which mediate tamoxifen-inducible Cre recombination specifically in endothelial cells [23] and subsequently with *ApoE* ^−/−^ mice. To induce Cre-mediated excision of the loxP-flanked p38α allele in endothelial cells, we fed groups of 6–8 week old *p38α^EC-KO^/ApoE^−/−^* and their *p38α^FL/FL^/ApoE^−/−^* littermates that did not carry the Tie2ER^T2^Cre transgene with a diet containing tamoxifen (400 mg/kg tamoxifen citrate, 5% sucrose in phytoestrogen-free chow) for 5 consecutive weeks [24]. The mice were subsequently placed on HCD for 10 weeks to facilitate the development of atherosclerotic plaques. To assess the efficiency of p38α ablation, we measured p38α expression in primary endothelial cells isolated by CD146-mediated magnetic cell sorting (MACS) from the lungs of mice, at the end of the HCD feeding. Flow cytometric analysis of cell fractions collected during MACS sorting showed efficient separation of CD146^+^ and CD146^−^ fractions (Figure S1). Immunoblot analysis of protein lysates from whole lung, CD146^+^ and CD146^−^ cell fractions showed efficient ablation of p38α in endothelial cell isolates from *p38α^EC-KO^/ApoE^−/−^* mice, indicating that tamoxifen-treatment induced efficient ablation of p38α in the vascular endothelium that persisted during the period of HCD feeding (Figure 2A).

**Figure 3:**
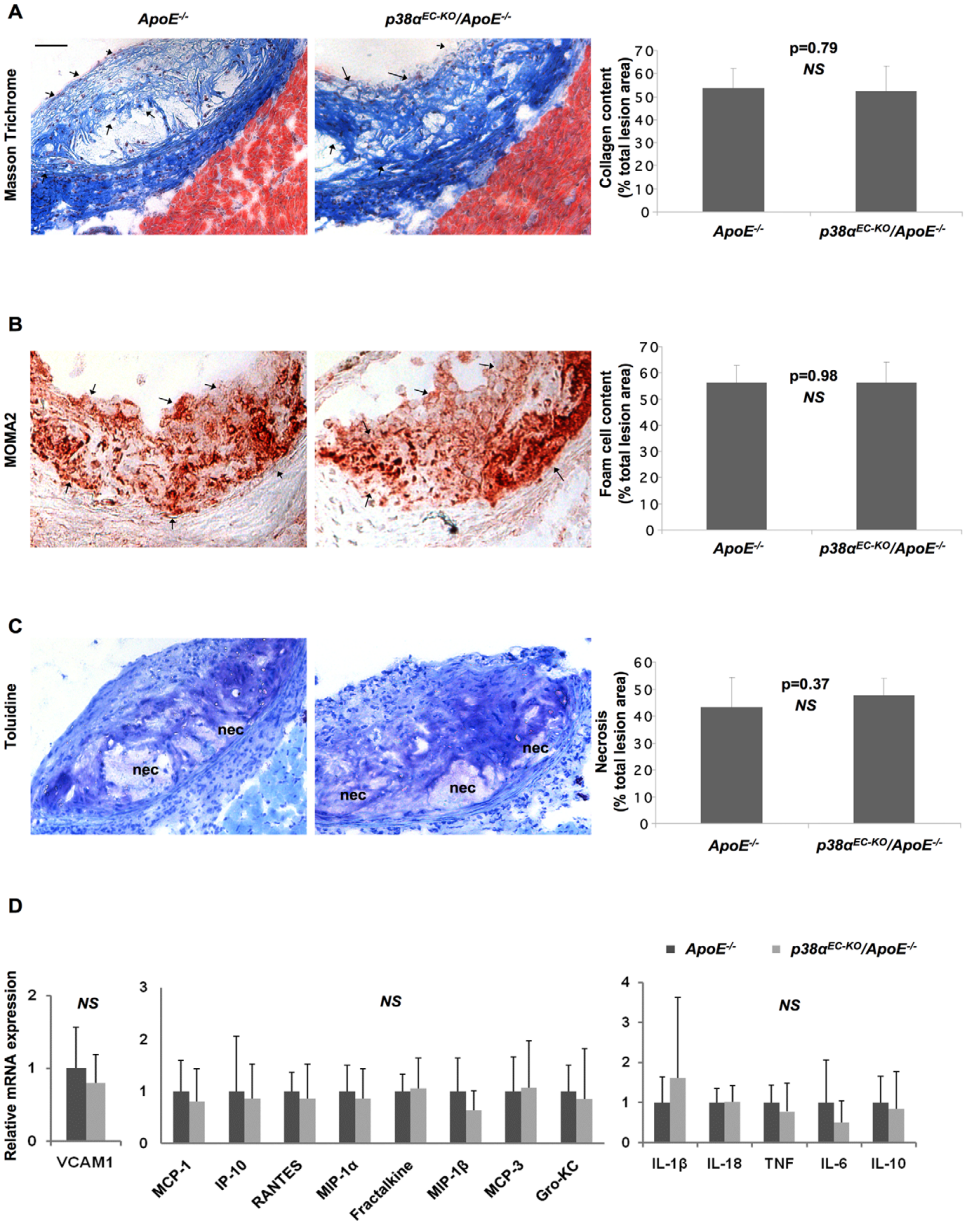
Similar plaque characteristics, cytokine, chemokine and adhesion molecule expression in *p38α^EC-KO^/ApoE^−/−^* and *ApoE^−/−^* mice. Staining and quantification at the aortic sinus of *p38α^EC–KO^/ApoE^−/−^* mice for: (A) Collagen content, Masson Trichrome staining (blue fibres, indicated by arrows) (B) Foam cell content, MOMA2 staining (red, indicated by arrows). (C) Necrotic core area, clear areas in lesions that did not stain for toluidine blue (nec = necrosis). (D) Relative mRNA levels of adhesion molecules, chemokines and pro-inflammatory cytokines (from left to right) of aortal arches from *p38α^EC-KO^/ApoE^−/−^* and *ApoE^−/−^* mice after 10 weeks on HCD. *P38α^EC-KO^/ApoE^−/−^* females, n = 9; *ApoE^−/−^* females, n = 6. Scale bar 50 µm. Error bars represent SD.

Measurement of bodyweight and cholesterol levels before and after the HCD treatment revealed no differences between *p38α^EC-KO^/ApoE^−/−^* and their *ApoE* ^−/−^ littermate controls, which showed similarly increased cholesterol levels and body weight after HCD feeding (Figure S2A). After 10 weeks on HCD, mice were sacrificed, and atherosclerotic lesion development was assessed in the whole aorta by *en face* staining with Sudan IV (Figure 2B), but also at the aortic sinus by histological analysis of consecutive sections followed by cross-sectional plaque area quantification (Figure 2C). This analysis did not reveal differences in lesion size between *p38α^EC-KO^/ApoE^−/−^* and their *ApoE^−/−^* littermates, either in the whole aorta or at the aortic sinuses. To further characterize the lesions, we assessed collagen and foam cell content and necrotic core formation. Quantification of collagen on sections stained with Masson Trichrome did not reveal differences in collagen content in plaques from *p38α^EC-KO^/ApoE^−/−^* mice compared to *ApoE* ^−/−^ controls (Figure 3A). In addition, staining with a MOMA2 antibody to detect macrophages showed a similar foam cell content in plaques from *p38α^EC-KO^/ApoE^−^* ^/−^ mice compared to *ApoE* ^−/−^ controls (Figure 3B). Finally, quantification of necrotic core area in sections from the aortic sinus did not reveal differences between *p38α^EC-KO^/ApoE^−/^* ^−^ and *ApoE* ^−/−^ controls (Figure 3C). Taken together, our experiments showed that endothelial p38α deficiency does not affect atherosclerotic plaque size or composition in *ApoE* ^−/−^ mice. These results were surprising in light of our *in vitro* findings, showing that p38α was essential for oxLDL-induced expression of adhesion molecules and chemokines that are important for monocyte recruitment in atherosclerosis. We therefore assessed the expression of a number of cytokines, chemokines and adhesion molecules in aortic roots from *p38α^EC-KO^/ApoE^−/−^* and *ApoE* ^−/−^ control mice at the end of the HCD treatment. These experiments did not show any significant differences in cytokine, chemokine or adhesion molecule expression between p38α^EC-KO^/*ApoE* ^−/−^ and *ApoE* ^−/−^ control mice (Figure 3D). Thus, our results show that endothelial cell-specific ablation of p38α does not affect the development of atherosclerosis in the *ApoE* ^−/−^ model of the disease.

### Macrophage-specific function of p38α in atherosclerosis

**Figure 4:**
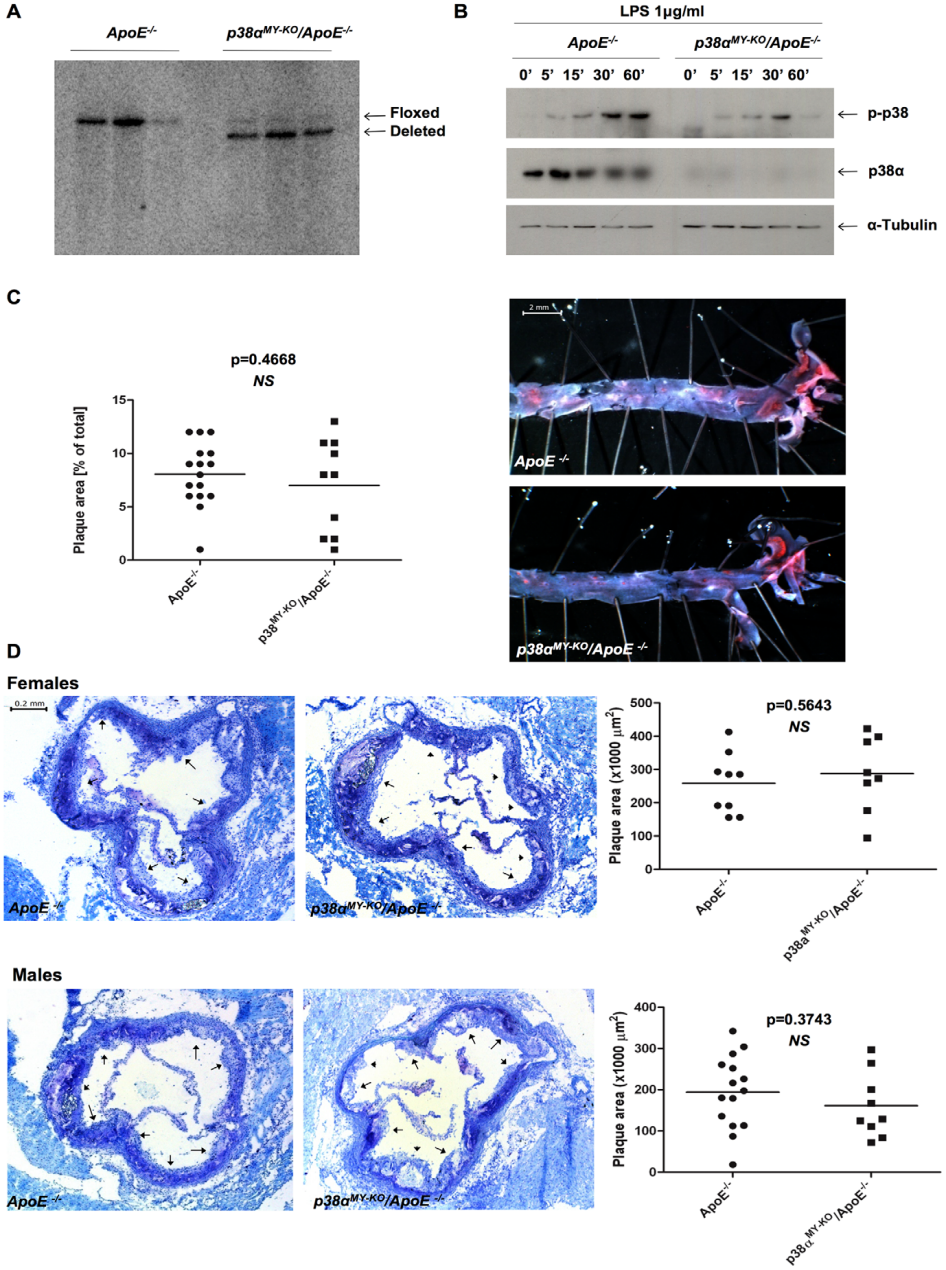
P38α ablation in macrophages does not affect atherosclerosis development. (A) Southern blotting on protein extracts from *p38α^MY-KO^/ApoE^−/−^* and *ApoE^−/−^* BMDMs to analyse p38α deletion efficiency in macrophages. P*38α^MY-KO^/ApoE^−/−^* mice, n = 3; *ApoE^−/−^*, n = 3. (B) LPS (1 µg/ml, Sigma) stimulation and immunoblotting of protein extracts from *p38α^MY-KO^/ApoE^−/−^* and *ApoE^−/−^* BMDMs. (C) Quantification of lesion size on whole aorta from male *p38α^MY-KO^/ApoE^−/−^* and *ApoE^−/−^* mice. *En face* Sudan IV staining of lesions. *P38α^MY-KO^/ApoE^−/−^* males, n = 9; *ApoE^−/−^* males, n = 9. Scale bar 2 mm. (D) Quantification of lesion area of atherosclerotic plaques at the aortic sinus of *p38α^MY-KO^/ApoE^−/−^* and *ApoE^−/−^* mice. Plaques are marked by arrows on aortal cross sections at the height of the aortic sinus. *P38α^MY-KO^/ApoE^−/−^* males, n = 9; *ApoE^−/−^* males, n = 15; *p38α^MY-KO^/ApoE^−/−^* females, n = 8; *ApoE^−/−^*, n = 9. Scale bar, 0.2 mm.

To study the macrophage-specific role of p38α in atherosclerosis, we crossed *p38α^FL/FL^/ApoE^−/−^* mice with LysMCre mice expressing Cre recombinase in macrophages and neutrophils [25]. Southern blot and immunoblot analysis of DNA and protein extracts from Bone Marrow- Derived Macrophage (BMDM) extracts showed efficient ablation of p38α in macrophages of *p38α^MY-KO^/ApoE^−/−^* mice (Figure 4A and B middle panel, respectively and figure S3A). LPS stimulation induced strong p38 phosphorylation in ApoE*^−/−^* control cells, while low levels of p38 phosphorylation were detected in p38α-deficient macrophages (Figure 4B, top panel and S3B).

**Figure 5:**
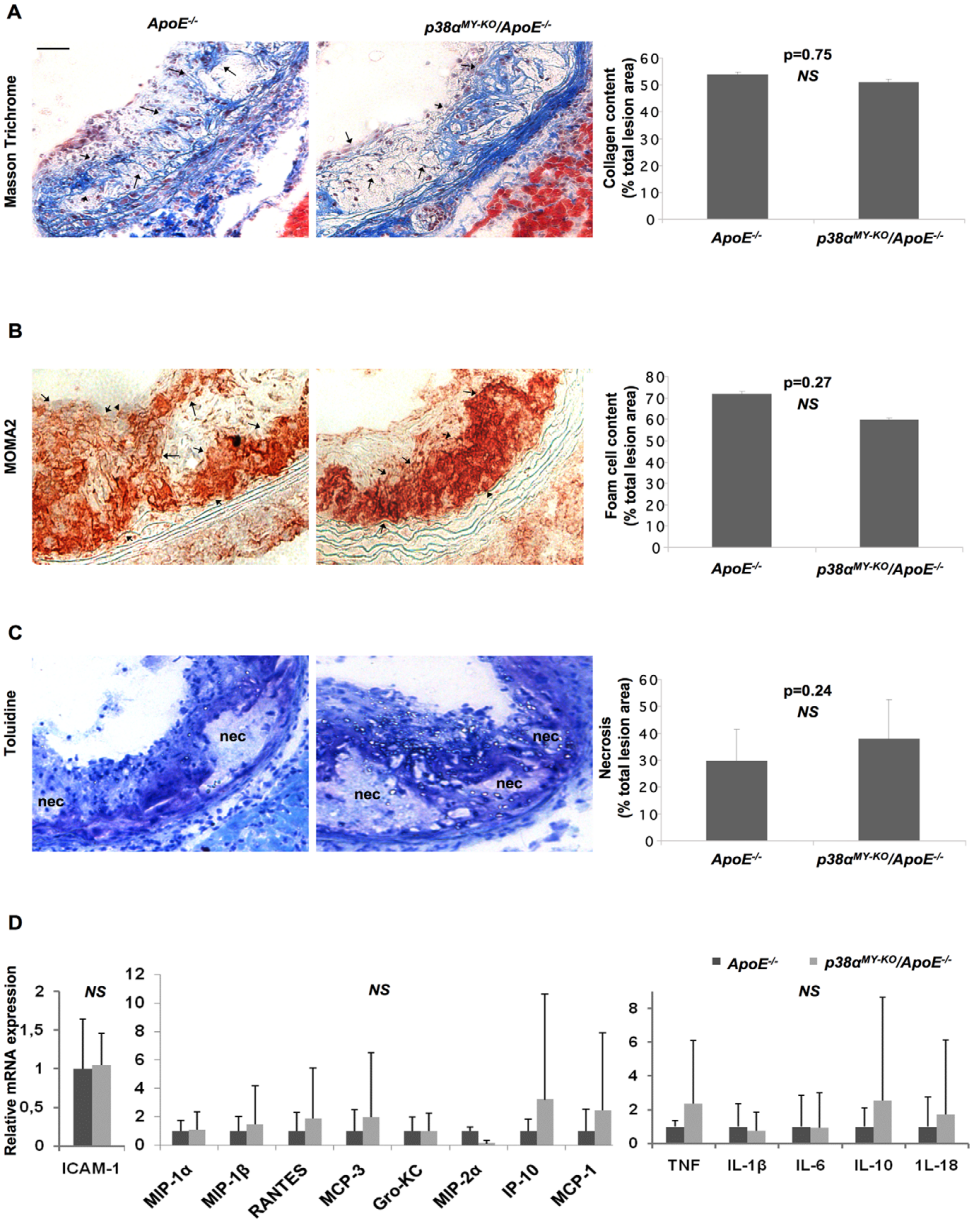
Similar plaque characteristics, cytokine, chemokine and adhesion molecule expression in *p38α^MY-KO^/ApoE^−/−^* and *ApoE^−/−^* mice. Staining and quantification at the aortic sinus of *p38α^MY-KO^/ApoE^−/−^* and *ApoE^−/−^* mice for: (A) Collagen content, Masson Trichrome staining (blue fibres, indicated by arrows), (B) Foam cell content, MOMA2 staining (red, indicated by arrows), (C) Necrotic core area, clear areas in lesions that did not stain for toluidine blue (nec = necrosis). (D) Relative mRNA levels of adhesion molecules, pro-inflammatory cytokines and chemokines (left to right) of aortal arches from *p38α^MY-KO^/ApoE^−/−^* and *ApoE^−/−^* mice after 10 weeks on HCD. *P38α^MY-KO^/ApoE^−/−^* females, n = 8; ApoE^−/−^ females, n = 9. Scale bar 50 µm. Error bars represent SD.

To address the potential role of macrophage p38α in atherosclerosis, we placed groups of male and female *p38^MY-KO^/ApoE^−/−^* and their *ApoE* ^−/−^ littermates on HCD for 10 weeks, starting from 6–8 weeks of age. Analysis of bodyweight and cholesterol levels, before and after the HCD treatment, revealed no differences between the two genotypes, which showed similarly increased cholesterol levels and body weight after HCD feeding (Figure S2B). After 10 weeks on HCD, mice were sacrificed, and atherosclerotic lesion development was assessed in the whole aorta by *en face* staining with Sudan IV (Figure 4C), but also at the aortic sinuses (Figure 4D). This analysis did not reveal differences in lesion size between *p38α^MY-KO^/ApoE^−/−^* and their *ApoE^−/−^* littermates, either in the whole aorta or in the aortic sinuses. Quantification of collagen content, foam cell formation and necrotic area also did not reveal any differences between the two genotypes (Figure 5A, B and C, respectively). Taken together, our experiments showed that macrophage p38α deficiency did not affect atherosclerotic plaque development in *ApoE* ^−/−^ mice as assessed by quantification of lesion size, collagen and macrophage content and necrotic core area. These findings are in contrast to the results reported by Seimon and colleagues, who found increased lesional necrosis and decreased collagen content in atherosclerotic plaques from *p38α^MY-KO^*/*ApoE* ^−/−^, compared to *ApoE* ^−/−^ controls [8].

Lesional macrophages secrete a wide range of pro-inflammatory and pro-atherosclerotic genes and p38α has been shown to regulate the expression of many of these genes in this cell type [18], [26], [20]. Thus, we measured the expression of a panel of cytokines, chemokines and adhesion molecules on RNA isolated from the aortic arch of *p38α^MY-KO^/ApoE^−/−^* and *ApoE* ^−/−^ littermates after 10 week on HCD. QRT-PCR expression analysis failed to reveal any considerable differences in the expression of any of the genes tested (Figure 5D), suggesting that p38α macrophage deficiency does not seem to significantly affect the expression of several pro-inflammatory mediators in the aorta.

Previous studies using chemical p38 inhibitors suggested that p38α activity in macrophages is required for oxLDL uptake and foam cell formation [12]. To address the potential role of p38α in oxLDL uptake we stimulated thioglycolate-elicited peritoneal macrophages (PMs) from *p38α^MY-KO^/ApoE^−/−^* and *ApoE* ^−/−^ mice with 50 µg/ml oxLDL and measured uptake by flow cytometric analysis (Figure S4A) and Oil Red O staining for lipids by microscopy (Figure S4B). Contrary to the findings of Zhao *et al.*, our results revealed that p38α deficiency did not affect the capacity of macrophages to take up oxLDL.

In conclusion, the results from our *in vivo* and *in vitro* analysis did not reveal differences in any of the examined parameters addressing atherosclerotic plaque formation in *p38α^MY-KO^/ApoE^−/−^* mice compared to *ApoE* ^−/−^ controls, suggesting that p38α signaling in macrophages does not play an important role in the development of atherosclerosis in the ApoE deficient mouse model.

## Discussion

Inflammation is now recognized as a critical pathogenic component for the development and progression of atherosclerotic plaques. Modified lipids act on resident vascular cells and also infiltrating inflammatory cells to induce and sustain the constant recruitment of blood monocytes into the arterial intima. Recruited monocytes differentiate into lipid laden foam cells, which are trapped and eventually die within the lesions. Inefficient clearance of apoptotic foam cells gives rise to a necrotic core in advanced lesions [5]. Thus, the mechanisms that control the recruitment, differentiation, activation, death and clearance of macrophages in atherosclerotic plaques are crucial for the development and progression of atherosclerosis [3], [4].

Intracellular signaling pathways that are activated downstream of innate and cytokine receptors and coordinate the expression of adhesion molecules, chemokines and cytokines that control inflammatory responses, are believed to play an important role in atherosclerosis. The p38 MAPK pathway is activated by a multitude of inflammatory and danger signals and coordinates cellular responses including survival, differentiation, proliferation, and the activation of pro-inflammatory gene expression programs [27]. These properties strongly suggested that p38 signaling must be implicated in the pathogenesis of atherosclerosis. In addition, systemic deficiency of MK2, a downstream effector of p38 signaling, reduced the severity of atherosclerosis in Ldlr^−/−^ mice [28], suggesting that p38 signaling indeed is important for this disease. We therefore decided to address *in vivo* the potential role of p38 in the development of atherosclerotic plaques. Since systemic p38α deficiency leads to embryonic lethality, we used conditional targeting of p38α in the *ApoE* ^−/−^ model of the disease. We chose to target p38α in endothelial cells and macrophages, two cell types with important functions in atherosclerosis.

Endothelial cell activation and expression of pro-inflammatory mediators and adhesion molecules plays a crucial role in the development of atherosclerosis by recruiting monocytes into the arterial intima [7]. OxLDL, cytokines but also shear stress can activate intracellular signaling pathways that control endothelial cell responses important for atherosclerosis. P38α has been implicated in this process through regulating migration and proliferation of endothelial cells [14], [15], cell permeability [16], apoptosis [17], expression of adhesion molecules like VCAM1 [18] and of various pro-inflammatory cytokines [10], [18]. Using p38α-deficient primary endothelial cells, we could indeed show here that p38α plays a crucial role in the expression of VCAM1 and the chemokines MCP-1, IP-10 and Gro-KC upon oxLDL stimulation. However, our *in vivo* experiments showed that endothelial cell-specific ablation of p38α did not affect the development of atherosclerosis in *ApoE* ^−/−^ mice, as assessed by measurement of lesion size, macrophage and collagen content and necrotic core area. In addition, endothelial p38α deficiency did not affect the expression of a number of proinflammatory mediators known to regulate atherosclerosis in aortic lesions from *ApoE* ^−/−^ mice fed with HCD for 10 weeks. Considering our *in vitro* results and the large amount of literature claiming an important role for p38α in endothelial cells for the development of atherosclerosis, the finding that endothelial p38α deficiency did not affect atherosclerosis development *in vivo* is surprising.

Likewise, myeloid cell specific p38α deficiency did not have a measurable effect in the development of atherosclerotic plaques in *ApoE* ^−/−^ mice fed for 10 weeks with a HCD, as assessed by *en face* staining of whole aortas and histological analysis of aortic root sections. Moreover, quantification of plaque characteristics like macrophage and collagen content and of necrotic core areas in aortic root lesions also revealed no considerable differences between *p38α^MY-KO^*/*ApoE* ^−/−^ and *ApoE* ^−/−^ mice. Inflammatory gene expression analysis in aortic lesions also failed to reveal an effect of macrophage p38α deficiency in the expression of mediators controlling the development of atherosclerosis. Thus, our results demonstrate that macrophage p38α deficiency did not affect the development of atherosclerotic plaques in the *ApoE* ^−/−^ mouse model, suggesting that macrophage p38α does not have an important role in the pathogenesis of atherosclerosis. Seimon *et al.* also studied the role of macrophage p38α in atherosclerosis using a similar experimental system [8]. In agreement with our results, they showed that macrophage p38α deficiency did not affect lesion size and macrophage content in aortic root plaques. However, in contrast to our findings and the findings from Jagavelu *et al.* with the downstream p38 effector MK2 [28], they found that plaques in *ApoE* ^−/−^ mice lacking p38α in macrophages showed increased necrotic areas, attributed to a function of p38α in protecting macrophages from ER-stress induced apoptosis. Since both studies were performed on mice in C57Bl/6 genetic background and using the same high cholesterol diet for a similar time period, currently, we cannot explain this difference between our study and that of Seimon *et al.*

At this stage we cannot exclude that combined deficiency of p38α in both macrophages and in endothelial cells might not affect atherosclerosis development *in vivo* or that p38α might function in other cell types such as smooth muscle cells to control atherosclerotic lesion pathogenesis. However, our findings highlight the potential complexity and redundancy in the processes regulating the development of atherosclerotic plaques *in vivo* and suggest that multiple mechanisms that are controlled by different signaling cascades contribute to the pathogenesis of atherosclerosis.

Taken together, our studies demonstrate that the macrophage and endothelial specific inhibition of p38α signaling does not protect against atherosclerosis. Without neglecting the fact that this molecule could be an important player in other cell types involved in atherosclerosis development (e.g. smooth muscle cells), these results suggest that p38 inhibitors may not be an appropriate therapeutic strategy for the prevention and treatment of atherosclerosis.

## Materials and Methods

### Ethics statement

All animal procedures were approved by the governmental ethics committee and were performed in compliance with the licenses No. /50.203.2-K13, 12/06/ of July, 7 2006 (elongated May, 12 2009) and /8.87-50.10.37.09.242/ of October, 10 2009, granted by the State Office for Nature, Environment and Consumer Protection of North Rhine-Westphalia (Landesamt für Natur, Umwelt und Verbraucherschutz Nordrhein-Westfalen), Germany. The investigation conforms to the ‘Guide for the Care and Use of Laboratory Animals’ published by the US National Institutes of Health (NIH Publication No. 85-23, revised 1996).

### Mice and diet

Conditional male and female *p38α^FL/FL^* [29] mice were crossed with *LysMCre* [25] and *Tie2ER^T2^Cre* [23] transgenic mice, to generate myeloid and endothelial cell-specific knockouts, respectively. These mice were then crossed with *ApoE^−/−^* mice [30], a widely accepted mouse model of atherosclerosis. For induction of Cre activity, mice carrying the *Tie2ER^T2^Cre* transgene and their Cre-negative littermates were fed a tamoxifen-containing diet (400 mg/kg tamoxifen citrate, 5% sucrose, 95% Teklad Global, 16% Rodent Diet) from Harland Teklad, as previously described [7], for 5 weeks. Atherosclerosis development was accelerated, in both macrophage- and endothelial-specific p38α knockout mice, by placing them on a high-cholesterol diet (HCD) from Harlan Teklad (Teklad Adjusted Calories 88137; 21% fat (WT/WT), 0.15% cholesterol (WT/WT) and 19.5% casein (WT/WT); no sodium cholate) for 10 weeks, starting at 6–8 weeks, or after tamoxifen feeding, respectively. All mice used in the experiments described here were backcrossed into the C57BL/6 genetic background for at least five generations.

### Genotyping and blood analysis

Primers for the wild-type, floxed or deleted p38α alleles were: *5′-CTACAGAATGCACCTCGGATG-3′*; *5′-AGAAGGCTGGATTTGCACAAG-3′*; *5′-CCAGCACTTGGAAGGCTATTC-3′* and for the wild-type or ApoE deleted alleles were: *5′-GCCTAGCCGAGGGAGAGCCG-3′*; *5′-TGTGACTTGGGAGCTCTGCAGC-3′*; *5′-GCCGCCCCGACTGCATCT-3′*. Primers for the LysMCre transgene were: *5′-CTTGGGCTGCCAGAATTTCTC-3′*; *5′-TTACAGTCGGCCAGGCTGAC-3′*; *5′-CCCAGAAATGCCAGATTACG-3′* and for the Tie2ER^T2^Cre transgene were: *5′-GTCCAATTTACTGACCGTACAC-3′*; *5′-CTGTCACTTGGTCGTGGCAGC-3′*.

Serum cholesterol level measurements were performed after overnight fasting using the PAD-CHOL reagent (Roche) and reading absorbance at 500 nm, according to manufacturer's instructions.

### Atherosclerotic lesion analysis

Consecutive 7 µm sections of the heart, at the level of the atrioventricular valves, were collected and stained with toluidine blue for morphometric analysis and atherosclerosis quantification, as previously described [7]. Toluidine stained sections were also used for the quantification of the necrotic core area, which was defined as a clear area that was toluidine free [8]. *En face* analysis of atherosclerotic lesions was performed by Sudan IV staining (red) of whole aortas, as described previously [7]. Plaque areas, in both the atrioventricular valves and the whole aortas, but also the necrotic core area, were quantified using Adobe Photoshop.

### Histology and immunostainings

For MOMA2 (anti-mouse macrophages/monocytes MCA519GT, Serotec) immunostainings, frozen sections of the aortic root were fixed in ice-cold acetone, blocked in 4% FCS with Avidin D solution (Avidin/Biotin Blocking Kit; Vector Laboratories) for 30 min and incubated with primary antibody (MOMA2, rat, 1∶1000) for 60 min in 4% FCS with Biotin (Avidin/Biotin Blocking Kit; Vector Laboratories). Sections were then incubated with biotin-conjugated rabbit anti-rat/biotinylated antibody (E0468, 1∶100, DakoCytomation) in 4%FCS/2% NMS for 60 min, incubated in Avidin/Biotin solution in PBS (Vectastain ABC Kit, Vector Laboratories) and color was developed with AEC substrate (Vector Laboratories). The sections were then counterstained with haematoxylin and mounted with Entellon (MERCK).

Immunostaining for collagen on frozen sections of the aortic root were performed by using the Masson Trichrome staining kit, which stains keratin and muscle fibers red, collagen green or blue, cytoplasm light red or pink, and cell nuclei dark brown to black (Sigma Aldrich), according to the manufacturer's instructions.

### Macrophage isolation

Bone marrow-derived macrophages (BMDM) were obtained according to standard procedures [7]. In brief, bone marrow cells isolated from the femurs of mice, were subjected to red blood cell (RBC) lysis and plated on 10 cm bacterial Petri dishes (Greiner) in RPMI Glutamax (Invitrogen) supplemented with 10% FCS, penicillin/streptomycin and 20% L929 conditioned medium. The cells were cultured for 8 days and starved in RPMI without FCS for 3 hrs, before experiments were performed.

Thioglycolate-elicited peritoneal macrophages (PM) were isolated as described elsewhere [31]. After RBC lysis, cells were washed extensively using ice-cold PBS and seeded on 6-well bacterial plates (Greiner) in RPMI Glutamax medium supplemented with 10% FCS. After 2 hrs, non-adherent cells were washed out with PBS and cells were starved for 48 hrs before experiments were performed.

### Mouse lung endothelial cell isolation (MLECs)

Lungs were dissected from PBS perfused mice, sterilized in 75% ethanol and placed in fresh DMEM (Gibco). Lungs were then cut into small pieces and incubated in 0.2% collagenase in DPBS+ CaCl_2_ (Gibco) for 1 hr at 37°C. Remaining tissue was broken down through a 19G needle and strained through a 70 µM cell strainer. The cell suspension was then centrifuged 1500 rpm/5 min and the pellet was resuspended in endothelial cell medium (DMEM low glucose: Ham's F-12 1∶1, 20% FCS, 50 µg/ml endothelial mitogen, 25 µg/ml heparin, 100 U/ml penicillin/streptomycin and 2 mM glutamine) and plated on a previously coated (0.1% gelatin, 10 µg/ml human fibronectin, 30 µg/ml bovine collagen type I) T75 flask. Endothelial cells were enriched by negative (LEAF rat anti-mouse CD16/32, 1∶2000 dilution, Biolegend) and positive (CD102 rat anti-mouse, 1.5∶2000 dilution, BD Biosciences) selection using Dynabeads (sheep anti-rat IgG, Invitrogen) during passages 1–2. Purity of the cell population was assessed by CD146 FITC (Miltenyi Biotec) staining and flow cytometric analysis.

At passage 8, MLECs were treated with His-TAT-NLS-Cre (HTNC) to induce recombination of p38α loxP flanked alleles [21], overnight (16 hrs), in medium containing DMEM (low glucose): RPMI: PBS (1∶1∶2 ratio) and 100 U/ml penicillin/streptomycin. The HTNC medium was then replaced with endothelial cell medium. The cells were allowed to reach confluence and passaged 1–2 times more before experiments were performed.

### MACS sorting of MLECS

MLECS were isolated from female *p38α^EC-KO^/ApoE^−/−^* and *ApoE^−/−^* littermates after a 10 week HCD. Collagenase dissociated lungs were passed through a 19G needle to form a cell suspension, which was subsequently passed through a 100 µm, a 70 µm and a 40 µm cell strainer. After washing twice with PBS, debris removal and cell enrichment of the remaining cell suspension was achieved by mixing with a 30% Histodenz solution (Sigma-Aldrich) and centrifuging 1500×g/2 mins to form a gradient. Low-density cells at the interface were harvested and washed twice in degassed MACS buffer (PBS pH 7.2, 0.5% BSA and 2 mM EDTA). Cells were resuspended in MACS buffer (90 µl/10^7^ cells) and labeled with Anti-LSEC magnetic MicroBeads (beads coupled to the mouse endothelial cell marker CD146, Miltenyi Biotec) by adding 10 µl beads/10^7^ cells and incubating 15 mins/4°C. After washing, the cells were resuspended in 500 µl buffer/10^8^ cells. Magnetic separation of the cells was performed on a MACS column (MS, Miltenyi Biotec), where CD146 positive endothelial cells were retained on the column and eluted only upon removal from the magnetic field. CD146 sorting of lung endothelial cells was confirmed through flow cytometry (FITC), after staining with a CD146-FITC conjugated antibody (Miltenyi Biotec), according to manufacturer's instructions. Cells obtained from MACS sorting were used to confirm efficient ablation of p38α in endothelial cells, by immunoblotting.

### Oxidized low-density lipoprotein stimulation (OxLDL)

CuSO_4_ oxidation of human LDL (AppliChem) was performed according to standard protocols [6]. DiI-labeling was achieved by further incubation of the oxidized products with 300 µg DiI reagent/mg LDL (Molecular Probes) for 18 hrs at 37°C, before dialysis.

OxLDL stimulation of PMs was performed as previously described, using 50 µg/ml DiI-labeled oxLDL [31]. Foam cell formation was visualized by staining overnight with Oil-red O (red) and counterstaining with hematoxylin. Uptake of oxLDL was quantified by flow cytometry (PE).

For MLECs, before oxLDL stimulation, cells were starved overnight unless otherwise indicated and 100 µg/ml oxLDL was added to the medium for indicated timepoints.

### Immunoblot analysis

Immunoblot analysis was performed as described elsewhere [29]. Membranes were probed with specific antibodies against α-tubulin (Sigma-Aldrich, T6074), p38α (Cell signaling, #9218), and p-p38 (Cell Signaling, #9211). Horseradish peroxidase-conjugated anti-rabbit and anti-mouse secondary antibodies were used (Amersham). Band volumetric analysis on western blots was performed using Radames software (Kindly provided by Dr. Hanns-Eugen Stöffler). P38α and total phosphorylated p38 were calculated by dividing the abundance of each protein by tubulin abundance (Figure S4).

### Southern blot analysis

Genomic DNA isolated from BMDM was digested overnight at 37°C in a 50 µl BamHI reaction (1×BamHI restriction buffer, 1 mM spermidine, 1 mM DTT, 100 µg/ml BSA, 50 µg/ml RNase A and 50 U BamHI enzyme/reaction). The DNA fragments were separated on a 0.8% agarose gel that was denatured by soaking for 45 mins in denaturing solution (0.4 M NaOH, 0.7% NaCl), and transferred overnight by capillary flow onto the surface of a charged nylon membrane (Hybond XL, Amerscham). The membrane was then soaked in neutralization solution (0.5 M Tris-HCl, 1.5 M NaCl), incubated at 70°C for 1 hr and prehybridized in hybridization buffer (1 M NaCl, 50 mM Tris-HCl pH 7.5, 10% dextransulphate, 1% SDS, 250 µg/ml Salmon Sperm DNA) at 65° for 3 hrs. DNA probes labeled by random priming with 32α-dGTP were added to the hybridization buffer and allowed to hybridize to the membrane overnight at 65°C. The membrane was then washed and exposed to autoradiographic film. Probe sequences used for the detection of p38α wild-type, floxed and deleted DNA fragments are available upon request.

### Quantitative real-time PCR

RNA was isolated from aortas, macrophages and endothelial cells using Trizol-reagent (Invitrogen) and RNeasy columns (QIAGEN), as previously described [7]. Briefly, RNA (1 µg) was used for reverse transcription with SuperScript III reverse transcriptase (Invitrogen). The reaction was topped up to 200 µl with water, and 2 µl were used for quantitative real-time PCR (qRT-PCR) reaction with the TaqMan qPCR Kit from Eurogentec. Standardization was performed with GAPDH (TaqMan). Taqman probes used for the quantification of MCP-1,VCAM1, IP-10, Gro-KC, RANTES, MIP-1α, Fractalkine, MIP-1β, MCP-3, IL-1β, IL-18, TNF, IL-6 and IL-10, were obtained from Applied Biosystems.

### Statistical analysis

All statistical analyses were performed using the Prism software (GraphPad Software Inc., San Diego, CA), and Excel. Statistical significance between experimental groups was assessed using an unpaired two sample Student's t test. Data are expressed as means ±SD. A p value of less than 0.05 is considered statistically significant.

## Supporting Information

Figure S1 **MACS sorting of MLECS.** Flow cytometric analysis of fractions collected during MACS sorting of lung endothelial cells from mice, after 10 weeks of HCD, showed efficient separation of CD146 positive and negative fractions.
(TIF)

Figure S2 **Cholesterol and bodyweight of mice on HCD.** Cholesterol (mg/dl) (top) and bodyweight (gr) (bottom) levels of (A) male and female *p38^EC-KO^/ApoE^−/−^* and *ApoE^−/−^* mice (*p38α^EC-KO^/ApoE^−/−^* males, n = 14; ApoE^−/−^ males, n = 15; *p38α^EC-KO^/ApoE^−/−^* females, n = 14; ApoE^−/−^ females, n = 13) and (B) male and female *p38α^MY-KO^/ApoE^−/−^* and *ApoE^−/−^* mice (*p38α^MY-KO^/ApoE^−/−^* males, n = 9; *ApoE^−/−^* males, n = 15; *p38α^MY-KO^/ApoE^−/−^* females, n = 8; *ApoE^−/−^* females, n = 9), before and after 10 weeks of a HCD. Error bars represent SD.
(TIF)

Figure S3 **Quantification of p38α expression and p38 phosphorylation in MLECs.** The expression of p38α and the levels of phosphorylated total p38 in figure 4B were quantified by performing volumetric analysis of the bands identified by immunoblot with p38α and p-p38 specific antibodies.
(TIF)

Figure S4 **Similar oxLDL uptake in** ***p38α^MY-KO^/ApoE^−/−^*** **and** ***ApoE^−/−^*** **macrophages, in vitro.** (A) Quantification by flow cytometry of oxLDL uptake by thioglycolate-elicited PMs, after 50 µg/ml DiI-oxLDL stimulation for 2.5 hrs. (B) Staining of oxLDL stimulated macrophages with Oil red O (lipid staining). Foam cells, defined as cells with ≥10 lipid droplets, are indicated with arrows. *ApoE^−/−^* mice, n = 3; *p38α^MY-KO^/ApoE^−/−^*, n = 3.
(TIF)
