# Supplementary figures and images for: Endothelial and Macrophage-Specific Deficiency of P38α MAPK Does Not Affect the Pathogenesis of Atherosclerosis in ApoE−/− Mice
Source: PLoS One. 2011 Jun 9;6(6):e21055. doi: 10.1371/journal.pone.0021055 (PMC3111465; doi:10.1371/journal.pone.0021055)

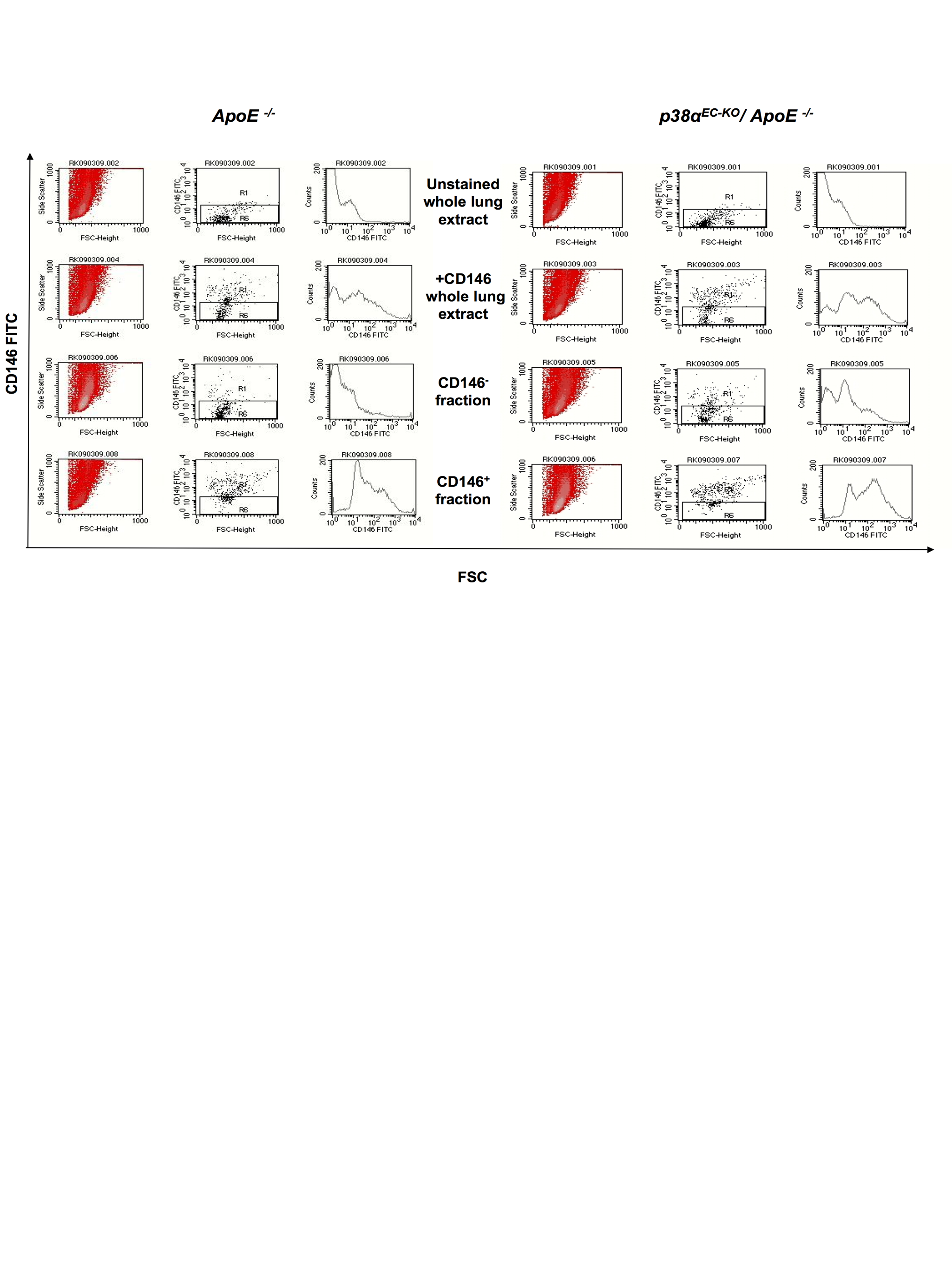

Supplement: Figure S1 — MACS sorting of MLECS. Flow cytometric analysis of fractions collected during MACS sorting of lung endothelial cells from mice, after 10 weeks of HCD, showed efficient separation of CD146 positive and negative fractions. (TIF) [file pone.0021055.s001.tif]

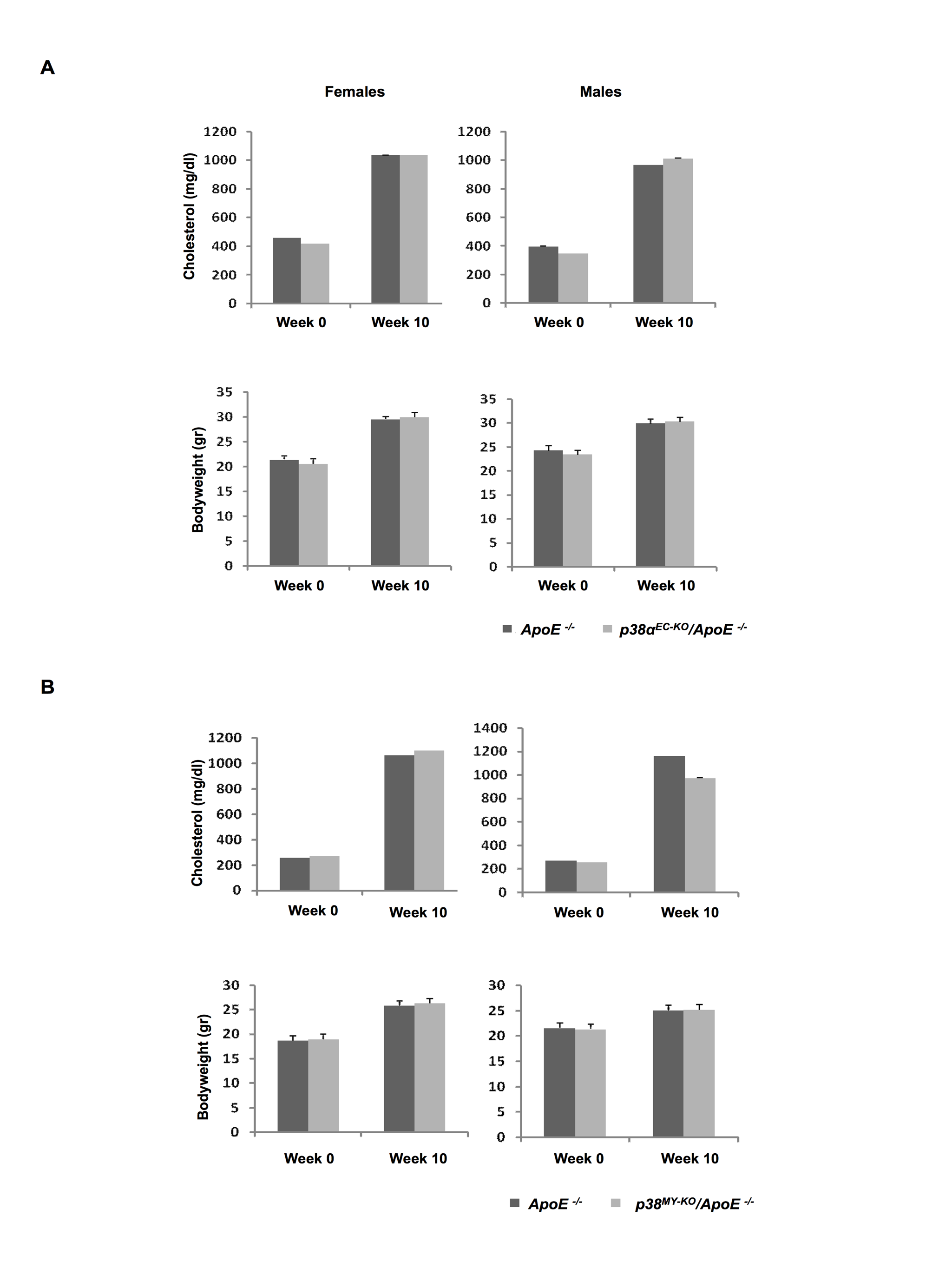

Supplement: Figure S2 — Cholesterol and bodyweight of mice on HCD. Cholesterol (mg/dl) (top) and bodyweight (gr) (bottom) levels of (A) male and female p38EC-KO/ApoE−/− and ApoE−/− mice (p38αEC-KO/ApoE−/− males, n = 14; ApoE−/− males, n = 15; p38αEC-KO/ApoE−/− females, n = 14; ApoE−/− females, n = 13) and (B) male and female p38αMY-KO/ApoE−/− and ApoE−/− mice (p38αMY-KO/ApoE−/− males, n = 9; ApoE−/− males, n = 15; p38αMY-KO/ApoE−/− females, n = 8; ApoE−/− females, n = 9), before and after 10 weeks of a HCD. Error bars represent SD. (TIF) [file pone.0021055.s002.tif]

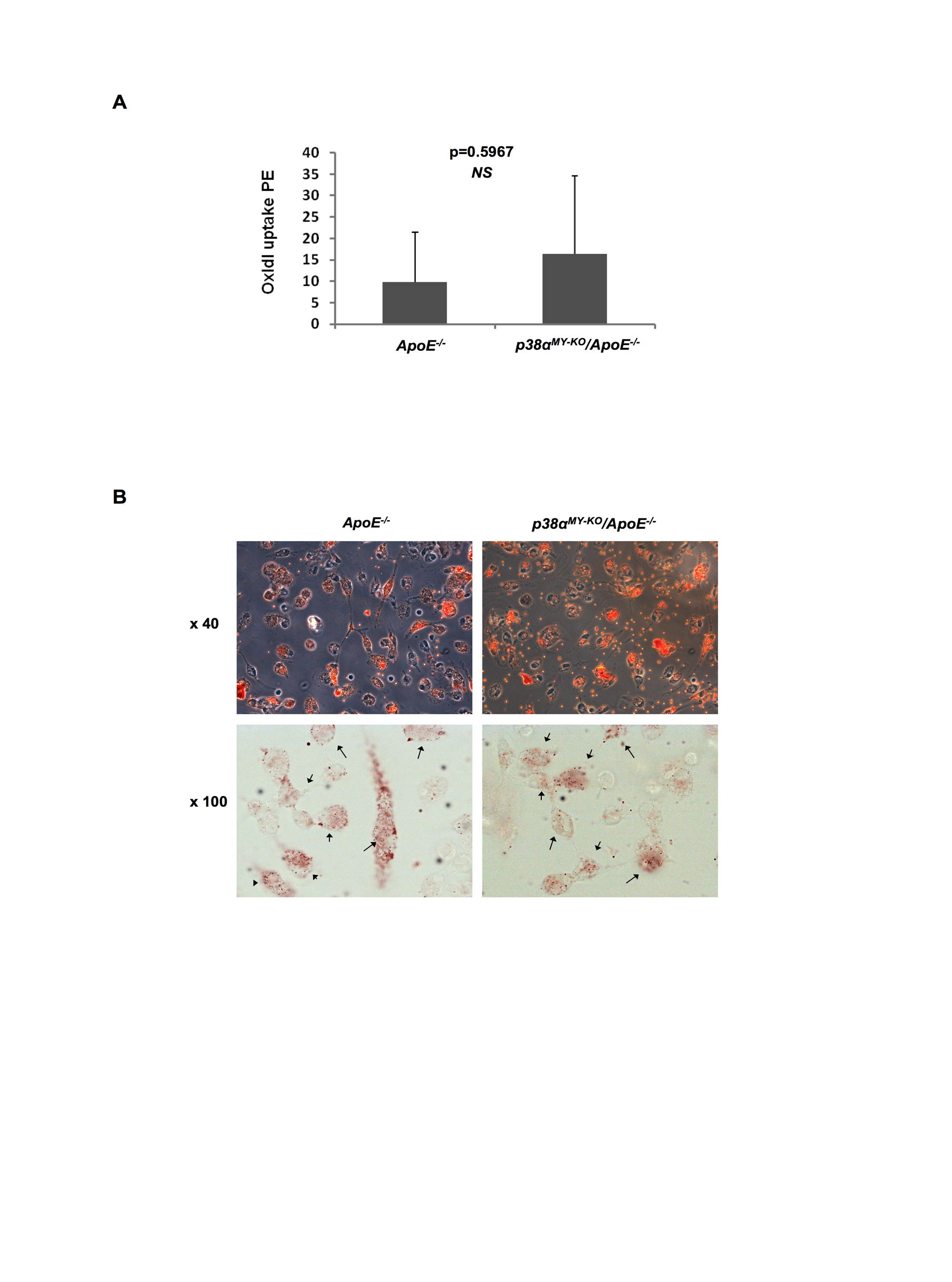

Supplement: Figure S4 — Similar oxLDL uptake in p38αMY-KO/ApoE−/− and ApoE−/− macrophages, in vitro. (A) Quantification by flow cytometry of oxLDL uptake by thioglycolate-elicited PMs, after 50 µg/ml DiI-oxLDL stimulation for 2.5 hrs. (B) Staining of oxLDL stimulated macrophages with Oil red O (lipid staining). Foam cells, defined as cells with ≥10 lipid droplets, are indicated with arrows. ApoE−/− mice, n = 3; p38αMY-KO/ApoE−/−, n = 3. (TIF) [file pone.0021055.s004.tif]
